# Tackling brain metastases from lung cancer during the COVID‐19 pandemic

**DOI:** 10.1002/cnr2.1276

**Published:** 2020-09-03

**Authors:** Naveen Mummudi, Anil Tibdewal, Tejpal Gupta, Vijay Patil, Kumar Prabhash, Jai Prakash Agarwal

**Affiliations:** ^1^ Department of Radiation Oncology, Tata Memorial Centre Homi Bhabha National Institute Mumbai India; ^2^ Department of Medical Oncology, Tata Memorial Centre Homi Bhabha National Institute Mumbai India

**Keywords:** brain metastases, COVID‐19 pandemic, lung cancer

## Abstract

Given the enormous strain the COVID‐19 pandemic has put on healthcare worldwide, appropriate allocation of resources according to priority is of immense importance. As brain metastases are a common presentation in lung cancer, during the pandemic, it potentially can pose a major management challenge to clinicians. In this article, we outline a pragmatic approach that oncologists should consider while managing these patients. The overarching principle is to deliver best, evidence‐based treatment without compromising patient care while ensuring the safety of healthcare workers.

## 1 INTRODUCTION

Lung cancer is the most commonly diagnosed cancer worldwide^1^ and a vast majority of patients present with locally advanced or metastatic disease.^2^ Almost 16% to 20% of non‐small cell lung cancer (NSCLC) patients develop brain metastases (BM) during the course of their treatment and around 10% present with upfront BM.^3^ The identification of oncogenic activation of tyrosine kinases in NSCLC, such as mutations in epidermal growth factor receptor (EGFR) or rearrangements of the anaplastic lymphoma kinase (ALK) gene, has enabled targeted molecular treatments which along with immunotherapy has brought about a paradigm change in the treatment landscape.

The management of patients with BM from lung cancer is, as such, quite diverse, challenging, and controversial.^4^ The pre‐existing health resource constraints in many countries have come under severe scrutiny due to the ongoing, unprecedented COVID‐19 pandemic, thereby compounding the management dilemma in various scenarios. Additionally, implementation of nationwide lockdown in a few countries and the ensuing travel limitations have resulted in restricted access to healthcare for patients.

As many countries near their peak infection rates, the decision to treat COVID‐19 positive patients should be weighed against the potential reduction in survival outcomes, if treatment is delayed or deferred. It is highly desirable that routine cancer care be resumed to the extent possible without compromising the health and safety of front‐line healthcare workers (HCWs). This may necessitate applying principles of resource allocation and priority settings.^5^ Some professional bodies and societies have published management guidelines and guiding principles for practicing neuro‐oncology during the pandemic^5^, ^6^, ^7^; few have focused on specific scenarios like management of gliomas,^8^, ^9^, ^10^ practice of radiosurgery,^11^, ^12^ and oncological emergencies.^13^ Guidelines have also been proposed for categorizing and managing patients with lung cancer during the pandemic.^14^, ^15^, ^16^ However, an elaborate and definitive guideline for management of BM during the pandemic is currently lacking. We outline practical considerations and propose pragmatic solutions for managing lung cancer patients with BM during the COVID‐19 pandemic. While the suggestions mentioned here are based on our recent institutional best practices, in the absence of evidence‐based management of cancer during global pandemics, we hope this document provides useful guidance to oncologists in attenuating the impact of this crisis on routine clinical care. We also recommend that clinicians consider these suggestions in accordance with existing institutional, state, and national regulations and policies as they evolve during the pandemic.

## 2 INITIAL SYMPTOM MANAGEMENT

- Anti‐edema measures
  - Patients presenting with symptoms and signs suggestive of raised intracranial tension pose an oncological emergency. Appropriate medical decompressive therapy has to be instituted in a timely manner, pandemic notwithstanding.
  - Steroid constitutes the staple of many neurological interventions and can effectively control symptoms by reducing peri‐tumoral edema. Initially, there were concerns regarding the use of steroids during the pandemic that it could potentially exacerbate respiratory symptoms and worsen outcomes. However, recent reports point toward a immunomodulatory role of steroids in the management of SARS‐CoV infection leading to positive outcomes.^17^
  - Routine use of steroids is not indicated in neurologically asymptomatic patients.
  - Symptomatic patients may be started on loading high dose followed by tapering dose over 2 weeks.^18^ Higher maintenance dose of steroids (16 mg/d) have not shown to be beneficial than low dose (4 mg/d) and is also associated with increased toxicity.^19^, ^20^, ^21^
  - Oral steroids have an excellent bio‐availability and are absorbed within 30 minutes of absorption. Hence, oral steroids may be preferred to minimize hospital visits. Although there are no studies comparing oral vs intravenous steroids in BM, evidence from management of bronchial asthma, optic neuritis and multiple sclerosis suggest that oral steroids are a practical and effective alternative.
  - Intravenous mannitol may be considered only in case of severe neurological symptoms when rapid reduction in is desired, such as impending tentorial herniation. Alternatives like oral glycerol and other osmotic diuretics are generally not recommended due to lack of efficacy.
- Anti‐epileptics drugs (AEDs)
  - Although the prophylactic use of anti‐epileptics has been shown to be of no benefit,^21^, ^22^ patients with carcinomatosis meningitis, lesions in epileptogenic cortical locations or with history of seizures should be strongly considered for AEDs.
  - In case of seizures, during the pandemic, due to travel restrictions, patients may have difficulty in accessing immediate medical attention. Given the relative safety of recent AEDs, they may be prophylactically initiated to reduce distress for both patient and healthcare providers.

## 3 GENERAL PRINCIPLES OF MANAGEMENT (SEE SUMMARY IN TABLE 1)

**TABLE 1:** Summary of recommendations for managing BM during the COVID‐19 pandemic

Diagnosis	Attempt biopsy from most accessible site. If long delay is expected—consider starting therapy without HPR with consent, if strong clinico‐radiological suspicion
Symptomatic management	Oral steroids can be safely used for medical decompression. Prophylactic anti‐epileptics in case of parenchymal lesions, especially if in areas of high epileptogenic potential
Surgery	Consider alternative treatment. Decompressive surgery in case of impending tentorial herniation, significant midline shift. To be done only in COVID dedicated OTs with all appropriate PPE by experienced specialist.
Radiation therapy	Single fraction stereotactic radio‐surgery is an alternative to surgery. Use FFF beam with appropriate energy to reduce treatment time. Upfront WBRT for patients with multiple lesions, uncontrolled primary, symptomatic and progressive disease Short course hypofractionated treatment to be preferred. To treat either in a dedicated machine or as last patient to avoid cross infection. Best supportive care alone in patients with poor performance status
Systemic therapy	In driver mutation positive patients, upfront oral targeted agents to be started when patient not a candidate for focal therapy and asymptomatic. Decision to initiate systemic chemotherapy to be tailored according to other prognostic features (see text). Immunotherapy may be used, but with caution.

Abbreviations: BM, brain metastases; HPR, histopathological report; OT, operation theatre; FFF, flattening filter free; WBRT, whole brain radiation therapy.

Appropriate personal protection equipment (PPE), hand hygiene, and social distancing should be strictly practiced. Virtual multidisciplinary meeting may be done, whenever feasible. Clear communication between the teams would also ensure smooth operation especially when multiple units are involved. Additionally, the following points are to be taken into consideration while deciding on treatment:

### 3.1 Establishing histopathological diagnosis

- All attempts should be made to obtain a histopathological confirmation before initiating treatment. However, if there is a considerable delay expected due to resource constraints during the pandemic or if the patient's symptoms require immediate treatment initiation, the same may be done without histological confirmation after obtaining an informed consent based on strong clinico‐radiological evidence.
- Unless contra‐indicated, biopsy should still be done at a later opportune time, since in addition to establishing a definitive diagnosis, it also yields important prognostic information.
- To minimize the risk to HCWs involved in the procedure, biopsy should be attempted from the most easily accessible site of disease. Complex invasive procedures should be performed either by or under the supervision of an experienced specialist.

### 3.2 Surgery

- Surgery for BM may be considered as a low priority during the pandemic to ration resources and also to minimize risk of cross‐infection to patient and healthcare workers.
- However, it may be considered as appropriate in patients with controlled primary disease, solitary lesion at locations which may otherwise cause significant neurological symptom and when non‐surgical treatment is not appropriate.
- In patients with BM causing significant midline shift or impending herniation, decompressive surgery may be considered.
- Surgery may also be delayed and considered for a later date, if an effective alternative systemic therapy is available immediately, to tide over the pandemic.
- When performed, neurosurgeries on COVID‐19 positive patients should be performed in dedicated operating theatres only and special care should be taken during procedure to avoid aerosol dispersal and avoid them from reaching nose and eyes with the use of appropriate PPE.

### 3.3 Radiation therapy

- Whole brain radiation therapy (WBRT):
  - Studies have clearly shown that short course WBRT schedules yield similar survival and local control as compared to longer courses.^23^, ^24^ However, with increased dose per fraction, neurocognitive toxicities are common with short course RT.^25^
  - Hence, during the pandemic, a dose of 20 Gy in 5 fractions would be pragmatic and preferred over other protracted schedules. Patients in whom survival outcomes are expected to be better, longer course (30 Gy in 10 fractions) could be utilized.
  - Although 12 Gy in 2 fractions (once a week) may be considered in patients with poor performance status, best supportive care and steroids alone should be discussed and strongly considered in these patients.^27^
  - If stereotactic radiosurgery (SRS) facility is unavailable or not feasible, a hypo‐fractionated boost of 10 to 15 Gy after WBRT may be considered in recursive partitioning analysis class 1 and 2 patients^26^ or in the post‐operative setting.
  - Clinical planning of WBRT without simulation may be considered when appropriate to reduce exposure to HCW.
  - Treatment techniques should preferably involve minimal complexity to reduce burden on an already constrained system, decrease treatment time, increase efficacy of treatment workflow and ensure safety of HCW involved in the treatment.
- Stereotactic treatment
  - SRS is an option for patients with favorable disease biology—oligo‐metastases and controlled extra‐cranial disease.
  - Single fraction schedules should be preferable over other fractionated schedules. Depending on the volume and proximity to other critical structures, total dose may be decided.
  - When SRS is planned, non‐invasive frameless techniques should be practiced, as their precision is comparable to invasive frames.^28^
  - Sanitization and proper disposal of the immobilization devices should be done, as they would be in close contact with patient's mouth/nose.
  - Linear accelerator with a flattening filter‐free beam of appropriate energy can be used to reduce the total treatment time.
- A dedicated machine may be used to treat COVID‐19 positive patients; when unavailable, they can be treated toward the end of the day, so that the machines can be sanitized adequately overnight.
- Focal therapy necessitates more frequent neuro‐imaging (to assess response and monitor toxicity), which in the present scenario could pose risk to patient due to repeated travel/visits to hospital. Elective neuro‐imaging may can be deferred to a later date or be performed in case of symptoms.

### 3.4 Systemic therapy

- Patients who harbour oncogenic driver mutations should receive appropriate oral tyrosine kinase inhibitors (TKIs).
- During the pandemic, patients, in whom the mutation status cannot be established or a delay is expected in test results, may be considered for oral TKIs, if:
  - The clinical profile is suggestive of a driver mutation positive NSCLC, for example, adenocarcinoma in a young, female gender, non‐smoker.
  - Patient is unsuitable for any other systemic therapy options due to poor performance status, lack of resources, and restricted access to care.
- Patients with BM who require systemic chemotherapy have a relatively inferior survival and thus may be at a relatively lower priority during the pandemic. However, an individualized decision may need to be taken, considering factors like age, comorbidities, performance status, and toxicity profile of the agent while deciding on initiation of systemic therapy. Priority may be given to patients who are treatment naïve vs those on second/third line chemotherapy.
- Although, there are isolated reports of COVID‐19 positive patients with NSCLC receiving immunotherapy,^29^ there is still uncertainty regarding its safety and caution should be exercised.^30^
- Patients with BM may also have other sites of metastases; attention should be given to prevent possible symptoms that may arise from these lesions.

## 4 SPECIFIC CASE SCENARIOS (SEE FIGURE 1)

**FIGURE 1:**
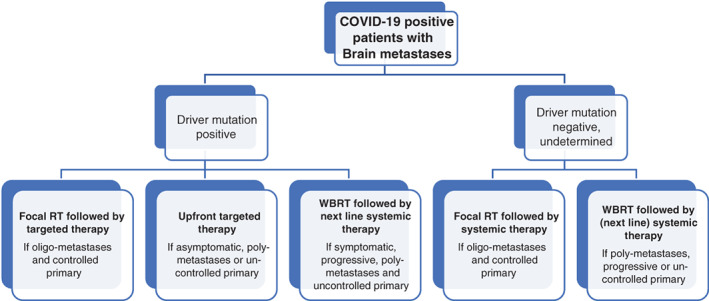
Algorithm for management of brain metastases (BM) in COVID‐19 positive patients

1. Oncogenic driver mutation (EGFR/ALK/ROS1) positive NSCLC

- Incidence of BM is higher in EGFR mutated and ALK rearranged NSCLCs; 24.4% at diagnosis and 46.7% at 3 years in EGFR‐mutated and 23.8% at diagnosis and 58.4% at 3 years in ALK‐rearranged NSCLCs.^31^
- In driver mutation positive patients, subsequent generation of inhibitors has significantly improved the progression free survival.^32^ Even during the pandemic, given the efficacy and relative safety of these newer agents, they should be considered as first choice in patients with BM who are treatment naïve and asymptomatic.
  - In the subset of patients who have solitary metastases, upfront local therapy may be considered.
  - In all other patients, for the period of pandemic, local therapy can be deferred or reviewed until after 3 months of systemic therapy or if patient develops new symptoms attributable to BM.
- Patient who are on second line TKIs who develop BM should be considered for agents which have better intracranial penetrance, if affordable and accessible. If BM are the only site of progression (oligo‐progression), local therapy (WBRT/SRS) can be given along with continuation of the same systemic agent.

- Oncogenic driver mutations negative NSCLC

- Asymptomatic, treatment naïve patients with oligo‐metastases may be candidates for SRS. However, during the pandemic, ENT situation, upfront initiation of systemic therapy might be preferable, except the group of patients with limited extra‐cranial disease which is also amenable for radical intent treatment.
  - Local therapy can be considered either at neuro‐progression with WBRT or in case of stable response/oligo‐progression with SRS at a later date.
- Asymptomatic patients with poly‐metastases may also be offered WBRT to pre‐empt symptomatic progression, if and when access to care becomes restricted.
- Patients with symptomatic, poly‐metastases, and active extra‐cranial disease should receive short course hypo‐fractionated WBRT.
- WBRT should also be offered to patients with progressive BM after first line or second line chemotherapy.

- Indeterminate oncogenic driver mutation status

- Patients with BM whose mutation status is yet to be established may be offered local therapy, while they await their test results.
  - Local therapy could be WBRT, if patient has poly‐metastases or extensive extra‐cranial disease.
  - SRS can be considered in patients with favorable disease profile (limited extra‐cranial disease, oligo‐metastases, or clinical history suggestive of driver mutation).

- Small cell lung cancer (SCLC)

- By definition, the presence of BM would frame a diagnosis of extensive stage SCLC. Symptomatic patients should receive short course WBRT followed by systemic chemotherapy. WBRT can be deferred in asymptomatic patients with small and few BM.

- Leptomeningeal disease

- Intrathecal methotrexate has uncertain benefit and is a resource intense treatment, which may be deferred during the pandemic. WBRT should be given only if there is co‐existing parenchymal BM.

## 5 CONCLUSION

BM in lung cancer is common and has a diverse range of clinical presentations. The COVID‐19 pandemic has put enormous constraint on existing healthcare infrastructure. Management of BM during this challenging time should take into account the safety of HCW along with the potential benefit patients may derive from proposed treatment. Depending on specific need of each patient, appropriate local and systemic therapy should be considered. As the pandemic continues to evolve, oncologists need to continually monitor the situation and adapt treatment decision as needed.

### ACKNOWLDEGEMENT

None.

### ETHICAL STATEMENT

Not Applicable.

## CONFLICT OF INTEREST

The authors have stated explicitly that there are no conflicts of interest in connection with this article.

## AUTHOR CONTRIBUTIONS

**Anil Tibdewal:** Writing‐review and editing. **Tejpal Gupta:** Supervision; visualization; writing‐original draft; writing‐review and editing. **Kumar Prabhash:** Writing‐original draft; writing‐review and editing. **Jai Prakash Agarwal:** Visualization; writing‐original draft; writing‐review and editing.

## Data Availability

Data sharing is not applicable to this article as no new data were created or analyzed in this study.
